# 6-Bromo-4-hydrazinyl­idene-1-methyl-3*H*-2λ^6^,1-benzothia­zine-2,2-dione

**DOI:** 10.1107/S1600536811027930

**Published:** 2011-07-23

**Authors:** Muhammad Shafiq, Islam Ullah Khan, Muhammad Zia-ur-Rehman, Muhammad Nadeem Arshad, Abdullah M. Asiri

**Affiliations:** a Materials Chemistry Laboratory, Department of Chemistry, GC University, Lahore 54000, Pakistan; b Applied Chemistry Research Center, PCSIR Laboratories Complex, Ferozpur Road, Lahore 54600, Pakistan; c X-ray Diffraction and Physical Laboratory, Department of Physics, School of Physical Sciences, University of the Punjab, Quaid-e-Azam Campus, Lahore 54590, Pakistan; d The Center of Excellence for Advanced Materials Research, King Abdul Aziz University, Jeddah, PO Box 80203, Saudi Arabia

## Abstract

In the title mol­ecule, C_9_H_10_BrN_3_O_2_S, the thia­zine ring has an envelope conformation with the S atom at the flap. The geometry around the S atom is distorted tetra­hedral. In the crystal, inversion dimers linked by pairs of N—H⋯N hydrogen bonds occur, generating *R*_2_^2^(6) ring motifs. N—H⋯O hydrogen bonds and C—H⋯O inter­actions connect the dimers, forming a three-dimentional network structure.

## Related literature

For the related structures of 6-bromo-1-methyl-1*H*-2,1-benzo­thia­zin-4(3*H*)-one 2,2-dioxide and 6-bromo-1-ethyl-1*H*-2,1-benzo­thia­zin-4(3*H*)-one 2,2-dioxide, see: Shafiq *et al.* (2009*a* ▶,*b* ▶), respectively. For the structures of other benzothia­zine derivatives, see: Shafiq *et al.* (2011 ▶); Arshad *et al.* (2011 ▶). For graph-set notation, see: Bernstein *et al.* (1995 ▶). For puckering parameters, see: Cremer & Pople (1975 ▶).
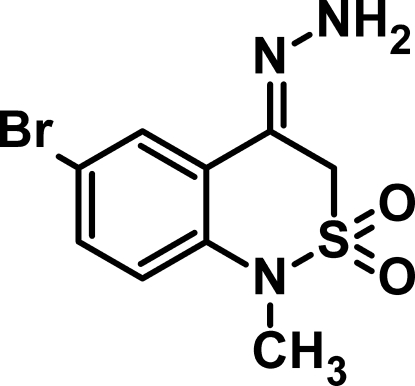


## Experimental

### 

#### Crystal data

C_9_H_10_BrN_3_O_2_S
*M* *_r_* = 304.17
Monoclinic, 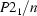

*a* = 10.1483 (5) Å
*b* = 9.6375 (4) Å
*c* = 11.2118 (5) Å
β = 92.278 (2)°
*V* = 1095.69 (9) Å^3^
*Z* = 4
Mo *K*α radiation
μ = 3.93 mm^−1^
*T* = 296 K
0.21 × 0.09 × 0.07 mm

#### Data collection

Bruker Kappa APEXII CCD diffractometer
Absorption correction: multi-scan (*SADABS*; Bruker, 2001 ▶) *T*_min_ = 0.492, *T*_max_ = 0.771
12176 measured reflections
2719 independent reflections
1972 reflections with *I* > 2σ(*I*)
*R*_int_ = 0.037

#### Refinement

*R*[*F* ^2^ > 2σ(*F* ^2^)] = 0.032
*wR*(*F* ^2^) = 0.079
*S* = 1.01
2719 reflections
152 parameters
H atoms treated by a mixture of independent and constrained refinement
Δρ_max_ = 0.40 e Å^−3^
Δρ_min_ = −0.35 e Å^−3^

### 

Data collection: *APEX2* (Bruker, 2007 ▶); cell refinement: *SAINT* (Bruker, 2007 ▶); data reduction: *SAINT*; program(s) used to solve structure: *SHELXS97* (Sheldrick, 2008 ▶); program(s) used to refine structure: *SHELXL97* (Sheldrick, 2008 ▶); molecular graphics: *ORTEP-3 for Windows* (Farrugia, 1997 ▶) and *PLATON* (Spek, 2009 ▶); software used to prepare material for publication: *WinGX* (Farrugia, 1999 ▶) and *PLATON*.

## Supplementary Material

Crystal structure: contains datablock(s) I, global. DOI: 10.1107/S1600536811027930/su2288sup1.cif

Structure factors: contains datablock(s) I. DOI: 10.1107/S1600536811027930/su2288Isup2.hkl

Supplementary material file. DOI: 10.1107/S1600536811027930/su2288Isup3.cml

Additional supplementary materials:  crystallographic information; 3D view; checkCIF report

## Figures and Tables

**Table 1:** Hydrogen-bond geometry (Å, °)

*D*—H⋯*A*	*D*—H	H⋯*A*	*D*⋯*A*	*D*—H⋯*A*
N3—H32⋯N2^i^	0.85 (4)	2.47 (4)	3.198 (4)	144 (3)
N3—H31⋯O1^ii^	0.90 (4)	2.38 (4)	3.252 (4)	162 (3)
C3—H3⋯O1^iii^	0.93	2.45	3.323 (3)	156

Symmetry codes: (i) 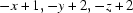; (ii) 
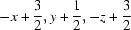
; (iii) 
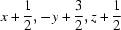
.

**Fig. 1.**
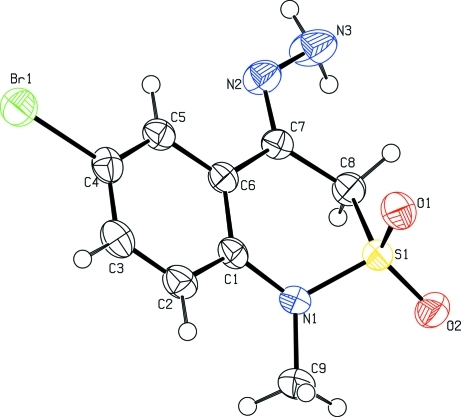
A view of the molecular structure of the title molecule, (I), showing the labelling scheme and 50% displacement ellipsoids.

**Fig. 2.**
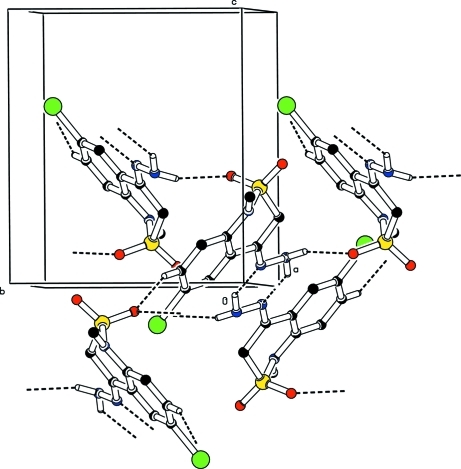
A perspective view of the crystal packing of compound (I), showing the inversion dimers formed through N—H···N hydrogen bonds (dashed lines; see Table 1 for details).

## supplementary crystallographic information

### Comment

Continuing our research on the synthesis (Shafiq *et al.*, 2011; Arshad *et al.*, 2011) and crystal structure studies of benzothiazine derivatives (Shafiq *et al.*, 2009*a*), we present herein the crystal structure of the title compound, (I).

The molecular structure of the title molecule, (I), is illustrated in Fig. 1. The structure differs to a similar published compound, 6-Bromo-1-methyl-1*H*-2,1-benzothiazin-4(3*H*)-one 2,2-dioxide (II) [Shafiq *et al.*, 2009*a*], in that the carbonyl group in (II) has been replaced with a hydrazide moiety in (I). The bond lengths and angles in the title compound are similar to those of (II) and in 6-Bromo-1-ethyl-1*H*-2,1-benzothiazin-4(3*H*)-one 2,2-dioxide (III) (Shafiq *et al.*, 2009*b*). In (I) atom Br1, attached to the planar aromatic ring (C1—C6), lies out of the plane by 0.0547 (3) Å, while in (II) and (III) the deviations are slightly greater, i.e. 0.064 (4) and 0.073 (4) Å, respectively. The thiazine ring, (C1/C6/C7/C8/S1/N1), has an envelope conformation with atom S1 as the flap [puckering parameters: Q (puckering amplitude) = 0.5873 (18) Å, θ = 124.31 (19) °, and φ = 185.9 (3) ° (Cromer & Pople, 1975)].

In the crystal structure of compound (I) the functional hydrazide group is involved in the formation of inversion dimers, through N3—H32···N2 hydrogen bonding, and generates a six-membered *R*_2_^2^(6) ring motif (Bernstein *et al.*, 1995). These dimers are further connected through N—H···O hydrogen bonds and weak C—H···O interactions to form a three dimensional network structure (Table 1, Fig. 2).

### Experimental

A mixture of 1-methyl-2,2-dioxo-2,3-dihydro-1H-2λ^6^-benzo [*c*][1,2]thiazin-4-one (10.60 g; 50.0 mmoles), hydrazine hydrate (85%) (5.0 ml) and ethanol (200 ml) was reacted at 318 K using an ultrasound reaction bath for about 35 mins. After completion of the reaction, excess hydrazine and solvent were removed under vacuum. The crude product obtained was washed with water and dried; Yield: 74%. Suitable crystals were produced through recrystalization in methanol under slow evaporation.

### Refinement

The NH H-atom was located in a difference Fourier map and refined with U_iso_(H)=1.2U_eq_(N). The C-bound H-atoms were included in calculated positions and treated as riding atoms: C-H = 0.93, 0.96, and 0.97 Å for CH(aromatic), CH_3_ and CH_2_ H-atoms, respectively, with U_iso_(H) = k × U_eq_(parent C-atom), where k = 1.5 for CH_3_ H-atoms and k = 1.2 for all other H-atoms.

### Crystal data

**Table:**

C_9_H_10_BrN_3_O_2_S	*F*(000) = 608
*M**_r_* = 304.17	*D*_x_ = 1.844 Mg m^−^^3^
Monoclinic, *P*2_1_/*n*	Mo *K*α radiation, λ = 0.71073 Å
Hall symbol: -P 2yn	Cell parameters from 3531 reflections
*a* = 10.1483 (5) Å	θ = 2.7–24.7°
*b* = 9.6375 (4) Å	µ = 3.93 mm^−^^1^
*c* = 11.2118 (5) Å	*T* = 296 K
β = 92.278 (2)°	Needle, yellow
*V* = 1095.69 (9) Å^3^	0.21 × 0.09 × 0.07 mm
*Z* = 4	

### Data collection

**Table:**

Bruker Kappa APEXII CCD diffractometer	2719 independent reflections
Radiation source: fine-focus sealed tube	1972 reflections with *I* > 2σ(*I*)
graphite	*R*_int_ = 0.037
φ and ω scans	θ_max_ = 28.3°, θ_min_ = 2.7°
Absorption correction: multi-scan (*SADABS*; Bruker, 2001)	*h* = −13→13
*T*_min_ = 0.492, *T*_max_ = 0.771	*k* = −12→7
12176 measured reflections	*l* = −14→14

### Refinement

**Table:**

Refinement on *F*^2^	Primary atom site location: structure-invariant direct methods
Least-squares matrix: full	Secondary atom site location: difference Fourier map
*R*[*F*^2^ > 2σ(*F*^2^)] = 0.032	Hydrogen site location: inferred from neighbouring sites
*wR*(*F*^2^) = 0.079	H atoms treated by a mixture of independent and constrained refinement
*S* = 1.01	*w* = 1/[σ^2^(*F*_o_^2^) + (0.0403*P*)^2^ + 0.1092*P*] where *P* = (*F*_o_^2^ + 2*F*_c_^2^)/3
2719 reflections	(Δ/σ)_max_ = 0.001
152 parameters	Δρ_max_ = 0.40 e Å^−^^3^
0 restraints	Δρ_min_ = −0.35 e Å^−^^3^

### Special details

**Table:**

Geometry. All s.u.'s (except the s.u. in the dihedral angle between two l.s. planes) are estimated using the full covariance matrix. The cell s.u.'s are taken into account individually in the estimation of s.u.'s in distances, angles and torsion angles; correlations between s.u.'s in cell parameters are only used when they are defined by crystal symmetry. An approximate (isotropic) treatment of cell s.u.'s is used for estimating s.u.'s involving l.s. planes.
Refinement. Refinement of F^2^ against ALL reflections. The weighted R-factor wR and goodness of fit S are based on F^2^, conventional R-factors R are based on F, with F set to zero for negative F^2^. The threshold expression of F^2^ > 2σ(F^2^) is used only for calculating R-factors(gt) etc. and is not relevant to the choice of reflections for refinement. R-factors based on F^2^ are statistically about twice as large as those based on F, and R- factors based on ALL data will be even larger.

### Fractional atomic coordinates and isotropic or equivalent isotropic displacement parameters (Å^2^)

**Table:**

	*x*	*y*	*z*	*U*_iso_*/*U*_eq_	
Br1	0.94817 (3)	0.62148 (3)	1.15166 (3)	0.04753 (12)	
S1	0.86913 (6)	1.05109 (6)	0.65175 (5)	0.03060 (16)	
O1	0.81377 (18)	0.92236 (19)	0.60884 (16)	0.0413 (4)	
O2	0.90132 (19)	1.15392 (19)	0.56664 (16)	0.0433 (5)	
N1	1.00013 (19)	1.0224 (2)	0.73776 (18)	0.0321 (5)	
N2	0.6567 (2)	1.0222 (2)	0.9307 (2)	0.0400 (5)	
N3	0.5556 (3)	1.1148 (3)	0.9019 (3)	0.0551 (7)	
H32	0.507 (4)	1.115 (3)	0.962 (3)	0.066*	
H31	0.586 (3)	1.199 (4)	0.881 (3)	0.066*	
C1	0.9838 (2)	0.9263 (2)	0.8314 (2)	0.0290 (5)	
C2	1.0852 (3)	0.8338 (3)	0.8633 (2)	0.0363 (6)	
H2	1.1616	0.8335	0.8202	0.044*	
C3	1.0743 (3)	0.7436 (3)	0.9567 (2)	0.0377 (6)	
H3	1.1427	0.6829	0.9774	0.045*	
C4	0.9602 (3)	0.7443 (2)	1.0195 (2)	0.0341 (6)	
C5	0.8576 (2)	0.8322 (2)	0.9895 (2)	0.0319 (6)	
H5	0.7813	0.8299	1.0328	0.038*	
C6	0.8669 (2)	0.9253 (2)	0.8943 (2)	0.0274 (5)	
C7	0.7567 (2)	1.0212 (2)	0.8646 (2)	0.0290 (5)	
C8	0.7637 (3)	1.1175 (2)	0.7582 (2)	0.0343 (6)	
H8A	0.7954	1.2077	0.7848	0.041*	
H8B	0.6761	1.1295	0.7219	0.041*	
C9	1.1299 (3)	1.0488 (3)	0.6900 (3)	0.0457 (7)	
H9A	1.1927	1.0658	0.7546	0.069*	
H9B	1.1250	1.1284	0.6385	0.069*	
H9C	1.1571	0.9694	0.6454	0.069*	

### Atomic displacement parameters (Å^2^)

**Table:**

	*U*^11^	*U*^22^	*U*^33^	*U*^12^	*U*^13^	*U*^23^
Br1	0.04682 (19)	0.04001 (17)	0.0552 (2)	−0.00435 (13)	−0.00552 (14)	0.01513 (13)
S1	0.0277 (3)	0.0349 (3)	0.0292 (3)	0.0003 (3)	0.0004 (3)	0.0005 (2)
O1	0.0432 (11)	0.0436 (10)	0.0366 (10)	−0.0068 (9)	−0.0041 (8)	−0.0078 (8)
O2	0.0411 (11)	0.0484 (11)	0.0406 (11)	0.0029 (9)	0.0046 (9)	0.0129 (8)
N1	0.0234 (11)	0.0400 (11)	0.0330 (12)	−0.0015 (9)	0.0008 (9)	0.0032 (9)
N2	0.0375 (13)	0.0384 (12)	0.0447 (14)	0.0089 (10)	0.0113 (11)	0.0073 (10)
N3	0.0439 (16)	0.0518 (16)	0.071 (2)	0.0180 (13)	0.0243 (14)	0.0177 (14)
C1	0.0265 (13)	0.0311 (12)	0.0290 (13)	−0.0005 (10)	−0.0034 (10)	−0.0058 (10)
C2	0.0283 (14)	0.0411 (14)	0.0394 (16)	0.0041 (11)	0.0000 (12)	−0.0047 (11)
C3	0.0337 (15)	0.0352 (13)	0.0436 (16)	0.0073 (11)	−0.0062 (12)	−0.0033 (11)
C4	0.0395 (15)	0.0257 (11)	0.0362 (14)	−0.0052 (10)	−0.0086 (12)	−0.0002 (10)
C5	0.0300 (14)	0.0319 (12)	0.0335 (14)	−0.0037 (10)	−0.0015 (11)	−0.0033 (10)
C6	0.0283 (13)	0.0259 (11)	0.0279 (13)	−0.0023 (10)	−0.0021 (10)	−0.0050 (9)
C7	0.0276 (13)	0.0293 (12)	0.0302 (13)	−0.0001 (10)	0.0022 (10)	−0.0035 (10)
C8	0.0322 (14)	0.0348 (13)	0.0360 (14)	0.0045 (11)	0.0028 (11)	0.0017 (11)
C9	0.0265 (14)	0.0676 (19)	0.0432 (16)	−0.0013 (13)	0.0049 (12)	0.0035 (14)

### Geometric parameters (Å, °)

**Table:**

Br1—C4	1.904 (2)	C2—H2	0.9300
S1—O2	1.4228 (19)	C3—C4	1.380 (4)
S1—O1	1.4369 (19)	C3—H3	0.9300
S1—N1	1.635 (2)	C4—C5	1.374 (3)
S1—C8	1.755 (3)	C5—C6	1.400 (3)
N1—C1	1.415 (3)	C5—H5	0.9300
N1—C9	1.464 (3)	C6—C7	1.479 (3)
N2—C7	1.280 (3)	C7—C8	1.515 (3)
N2—N3	1.388 (3)	C8—H8A	0.9700
N3—H32	0.85 (4)	C8—H8B	0.9700
N3—H31	0.90 (4)	C9—H9A	0.9600
C1—C2	1.397 (4)	C9—H9B	0.9600
C1—C6	1.404 (3)	C9—H9C	0.9600
C2—C3	1.369 (4)		
			
O2—S1—O1	118.29 (11)	C5—C4—Br1	120.2 (2)
O2—S1—N1	108.09 (11)	C3—C4—Br1	118.40 (19)
O1—S1—N1	110.45 (11)	C4—C5—C6	120.6 (2)
O2—S1—C8	111.38 (11)	C4—C5—H5	119.7
O1—S1—C8	107.55 (12)	C6—C5—H5	119.7
N1—S1—C8	99.44 (11)	C5—C6—C1	118.1 (2)
C1—N1—C9	121.3 (2)	C5—C6—C7	120.0 (2)
C1—N1—S1	115.59 (16)	C1—C6—C7	121.9 (2)
C9—N1—S1	118.49 (17)	N2—C7—C6	118.9 (2)
C7—N2—N3	117.8 (2)	N2—C7—C8	120.9 (2)
N2—N3—H32	105 (2)	C6—C7—C8	120.1 (2)
N2—N3—H31	112 (2)	C7—C8—S1	111.17 (16)
H32—N3—H31	115 (3)	C7—C8—H8A	109.4
C2—C1—C6	119.7 (2)	S1—C8—H8A	109.4
C2—C1—N1	120.0 (2)	C7—C8—H8B	109.4
C6—C1—N1	120.2 (2)	S1—C8—H8B	109.4
C3—C2—C1	121.4 (2)	H8A—C8—H8B	108.0
C3—C2—H2	119.3	N1—C9—H9A	109.5
C1—C2—H2	119.3	N1—C9—H9B	109.5
C2—C3—C4	118.8 (2)	H9A—C9—H9B	109.5
C2—C3—H3	120.6	N1—C9—H9C	109.5
C4—C3—H3	120.6	H9A—C9—H9C	109.5
C5—C4—C3	121.4 (2)	H9B—C9—H9C	109.5
			
O2—S1—N1—C1	177.43 (17)	C4—C5—C6—C1	−0.3 (3)
O1—S1—N1—C1	−51.7 (2)	C4—C5—C6—C7	−178.8 (2)
C8—S1—N1—C1	61.13 (19)	C2—C1—C6—C5	1.4 (3)
O2—S1—N1—C9	−26.3 (2)	N1—C1—C6—C5	−177.3 (2)
O1—S1—N1—C9	104.5 (2)	C2—C1—C6—C7	179.8 (2)
C8—S1—N1—C9	−142.6 (2)	N1—C1—C6—C7	1.1 (3)
C9—N1—C1—C2	−13.4 (3)	N3—N2—C7—C6	179.0 (2)
S1—N1—C1—C2	142.1 (2)	N3—N2—C7—C8	−0.3 (4)
C9—N1—C1—C6	165.3 (2)	C5—C6—C7—N2	3.4 (3)
S1—N1—C1—C6	−39.2 (3)	C1—C6—C7—N2	−174.9 (2)
C6—C1—C2—C3	−1.4 (4)	C5—C6—C7—C8	−177.2 (2)
N1—C1—C2—C3	177.3 (2)	C1—C6—C7—C8	4.4 (3)
C1—C2—C3—C4	0.4 (4)	N2—C7—C8—S1	−155.8 (2)
C2—C3—C4—C5	0.6 (4)	C6—C7—C8—S1	24.9 (3)
C2—C3—C4—Br1	−178.47 (19)	O2—S1—C8—C7	−165.88 (17)
C3—C4—C5—C6	−0.7 (4)	O1—S1—C8—C7	63.0 (2)
Br1—C4—C5—C6	178.42 (17)	N1—S1—C8—C7	−52.11 (19)

### Hydrogen-bond geometry (Å, °)

**Table:**

*D*—H···*A*	*D*—H	H···*A*	*D*···*A*	*D*—H···*A*
N3—H32···N2^i^	0.85 (4)	2.47 (4)	3.198 (4)	144 (3)
N3—H31···O1^ii^	0.90 (4)	2.38 (4)	3.252 (4)	162 (3)
C3—H3···O1^iii^	0.93	2.45	3.323 (3)	156.

Symmetry codes: (i) −*x*+1, −*y*+2, −*z*+2; (ii) −*x*+3/2, *y*+1/2, −*z*+3/2; (iii) *x*+1/2, −*y*+3/2, *z*+1/2.
